# Functional changes of the myocardium in survivors of high-voltage electrical injury

**DOI:** 10.1186/cc12506

**Published:** 2013-02-07

**Authors:** Kyoung-Ha Park, Sang Jin Han, Hyun-Sook Kim, Sang Ho Jo, Sung-Ai Kim, Suk-Won Choi, Seong Hwan Kim, Woo Jung Park

**Affiliations:** 1 Department of Cardiology, Hallym University Sacred Heart hospital, 896, Pyeongchon-dong, Dongan-gu, Anyang-si, Gyeonggi-do, 431-070, Korea; 2 Department of Cardiology, Korea University Ansan Hospital, 123, Jeokgeum-ro, Ansan-si, Gyeonggi-do, 425-707, Korea

## Abstract

**Introduction:**

There are limited long-term follow-up data on functional changes in the myocardium after high-voltage electrical injury (HVEI).

**Methods:**

Twenty-three patients who had been exposed to HVEI (>20,000 volts) and preserved left ventricular ejection fraction (≥55%) were enrolled in the study. Echocardiographic parameters, including peak systolic strain (S) and strain rate (SR), were evaluated at baseline, six weeks and six months later. These data were compared with a healthy control group who were matched in terms of age, sex and body mass index.

**Results:**

The systolic and diastolic blood pressure and the heart rate were significantly higher in the HVEI group compared with the control group at baseline and at six weeks, but not at the six-month follow-up. Conventional echocardiographic data showed no differences between the groups during the study period. In contrast to the S, the baseline and six weeks, SR was significantly increased in the HVEI group compared with the control group. However, at the six-month follow-up, there was no difference in the SR between the groups. Among the 23 patients with HVEI, 17 of the patients had vertical current injury, and 6 patients had horizontal current injury. There was no difference in terms of the conventional echocardiography, S and SR between the patients with vertical injury and those with horizontal injury at baseline and at the six-month follow-up.

**Conclusions:**

The long-term contractile performance of the myocardium is preserved when patient do not experience left ventricular dysfunction in the early stages after HVEI.

## Introduction

High-voltage electrical injury (HVEI) is relatively infrequent and various incidences of cardiac abnormalities after this type of injury have been reported [1,2]. Cardiovascular effects, including serious cardiac arrhythmia, myocardial damage and cardiac arrest, usually require close evaluations after HVEI [3]. The severity of the myocardial damage might depend on the voltage, the type of current, the duration of contact with the source and the pathway of the current in the patient's body [4,5]. Diagnosis of myocardial injury after HVEI is not easy because of the absence of typical chest pain, the lack of specific changes on electrocardiography (ECG) and the paucity of evidence supporting the utility of creatine kinase MB (CK-MB) levels.

Thus far, there have been a small number of echocardiographic studies of changes in myocardial function after HVEI with a relatively small sample size [6]. Therefore, it is not clear whether HVEI is associated with left ventricular (LV) dysfunction. Although the left ventricular ejection fraction (LVEF) is the current standard for measuring systolic function, a tissue Doppler image measures the strain (S) and the strain rate (SR), both of which are basic descriptors of the nature and the function of cardiac tissue and they have been applied to the assessment of LV function [7]. We have demonstrated with echocardiographic data using the S and SR that further deterioration of the LV function in survivors following HVEI with preserved LV function is uncommon [8]. However, the duration of follow-up was clearly short (seven days), and whether different results would be obtained with an examination at a long-term follow-up is unclear. We sought to examine changes in myocardial function using 2D speckle tracking imaging in patients with preserved LV function after HVEI in a long-term follow-up study.

## Materials and methods

### Study population

Patients were eligible for enrollment in this study if they were between 18 and 65 years old and had been exposed to a HVEI of more than 20,000 volts. Patients were excluded if they had been injured more than 48 hours before the study, had any cardiac disease, had left ventricular dysfunction (LVEF <55%), fatal arrhythmia or atrial fibrillation, serious external wounds on the left anterior chest wall, had sepsis or systemic shock, or were unable to follow the protocol. In addition, we enrolled healthy subjects matched in terms of age, sex and body mass index (control group) and compared the data of the patients with HVEI.

### ECG and blood samples for myocardial injury

Serial assessments using a standard 12-lead ECG (MAC 5000, GE Healthcare, Horten, Norway) were performed on the day of admission, six weeks and six months after the injury. Creatinine kinase (CK), CK-MB and cardiac troponin I (cTnI) levels in blood samples were obtained at admission and every 8 hours for the first 24 hours after admission until peak levels were measured.

### Conventional echocardiography and 2D speckle tracking imaging analysis

**Figure 1:**
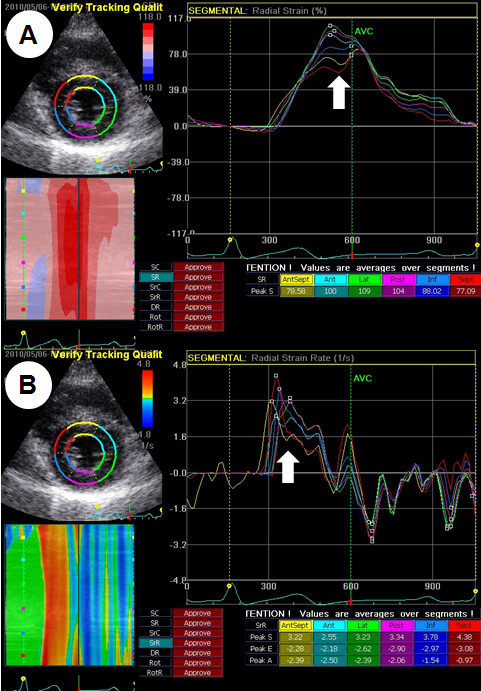
**Strain on the 2D speckle tracking image**. An example of speckle tracking radial strain (S, **A**) and the strain rate (SR, **B**) using the midventricular short-axis view. The LV myocardium was divided into six segments, which were color coded, and the values of the deformation parameters were displayed graphically for all six segments. The peak radial S and SR were obtained from the peak values of the systolic curves in each segment (arrow).

Conventional echocardiographic examinations were performed according to the American Society of Echocardiography (ASE) guidelines with a commercially available echocardiographic system (Vivid 7, GE Healthcare, Horten, Norway) on the day of admission, six weeks and six months later [9]. The tissue Doppler-derived early diastolic (Ea) velocity was measured at the mitral septal annulus. Two-dimensional (2D) gray-scale imaging (frame rate 40 to 80/sec) was performed in the apical four-chamber, apical two-chamber, apical three-chamber and mid-ventricular parasternal short-axis views. All images were digitally acquired and recorded for off-line analysis (EchoPac, GE Healthcare, Horten, Norway). The longitudinal S and SR were measured in all 16 myocardial segments. In addition, the circumferential and radial S and SR were obtained in the six segments of the mid-ventricular short-axis view. The endocardial borders were traced at the end-systolic frame, and an automated tracking algorithm outlined the myocardium in successive frames throughout the cardiac cycle. The tracking quality (adequate or inadequate) was estimated for each myocardial segment. In cases where the tracking quality was inadequate, the regions of interest were further adjusted manually to achieve adequate tracking quality for different segments. After the observer accepted the tracking quality, myocardial motion was evaluated by tracking speckles in the 2D gray-scale image. The LV myocardium was divided into six segments, which were color coded, and the values of the deformation parameters were displayed graphically for all six segments in each view. The myocardial SR values were obtained from the peak values of the systolic curves (peak systolic SR), and the S values were measured at end systole on the curves (peak systolic S) in each segment. The longitudinal S and SR values were obtained by averaging 16 myocardial segment values, and the circumferential and radial S and SR were evaluated based on the mean value of the six segments from the short-axis view (Figure 1). One cardiologist (SWC), blinded to the participants' clinical data, interpreted the echocardiogram using an off-line analysis. Intraobserver reproducibility of the S and SR were evaluated. The correlation coefficients for the longitudinal, circumferential and radial S and SR were 0.80 and 0.81 for longitudinal S and SR, 0.89 and 0.82 for circumferential S and SR, and 0.88 and 0.90 for radial S and SR, respectively (all *P *<0.01).

### Statistical analysis

Data are presented as mean ± SD. Categorical variables were presented as numbers or percentages. Comparisons of the clinical and the echocardiographic variables, including the myocardial S and SR values were made between the HVEI group and the control group using either an independent sample *t*-test or a Mann-Whitney *U *test. Differences in the categorical variables between the two groups were analyzed with the Chi-square test or Fisher's exact test. A value of *P *<0.05 indicated statistical significance. In addition, the patients with HVEI were divided according to the pathway of the current in the patient's body (vertical injury vs. horizontal injury), and their echocardiographic data were compared. All the variables of the control group were measured one time and compared with the baseline, six-week and six-month follow-up data of the HVEI group. This study was approved by the Institutional Review Board of the Hallym University Medical Center and all patients gave their written informed consent.

## Results

Between May 2010 and September 2011, 64 survivors of HVEI were assessed. Among these, 41 patients were excluded for the following reasons: admitted more than 48 hours after the event (*n *= 17), LV dysfunction (*n *= 3), atrial fibrillation (*n *= 1), combined serious flame burn wound on left anterior chest wall (*n *= 6), multi-organ failure or sepsis (*n *= 9), or refusal to participate in the study (*n *= 5). Among the remaining 23 patients with HVEI, 17 had vertical current injury from the entrance to the exit sites, and six patients exhibited horizontal current injury. The average burn size due to HVEI was 6.3%. The mean level of CK was 10,365 ± 13,839 IU/l, CK-MB was 88.1 ± 108.2 ng/ml, and cTnI was 0.086 ± 0.110 ng/ml. During the study, two patients did not undergo the six-month echocardiography study due to follow-up loss.

### Baseline patient characteristics

No significant differences were noted between the HVEI group and the control group in terms of their baseline clinical characteristics including age, cardiovascular risk factors, body mass index and medications used, except for elevated fasting glucose and high-sensitive C-reactive protein in the HVEI group (Table 1).

**Table 1:** Baseline clinical characteristics and medications

	HVEI group (*n *= 23)	Control group (*n *= 23)	*P*-value
Age (years)	49 ± 11	49 ± 11	0.95
Hypertension	5 (21.7%)	6 (26.1%)	0.73
Current smoking	13 (56.5%)	8 (34.8%)	0.14
Diabetes	1(4.3%)	0 (0%)	1.00
Dyslipidemia	3 (13.0%)	3 (13.0%)	1.00
Body mass index (Kg/m^2^)	23.9 ± 2.4	23.5 ± 2.0	0.55
Hemoglobin (gm/dl)	14.7 ± 1.0	15.1 ± 1.2	0.16
Fasting glucose (mg/dl)	111 ± 18	92 ± 10	<0.01
Total cholesterol (mg/dl)	174 ± 27	184 ± 26	0.22
Creatinine (mg/dl)	0.8 ± 0.1	0.8 ± 0.1	0.65
High-sensitive C-reactive protein (mg/dl)	48.5 ± 38.5	0.8 ± 0.6	<0.01

Beta-blocker	1 (4.3%)	2 (8.7%)	1.00
ACEI/ARB	2 (8.7%)	4 (17.4%)	0.67
Calcium channel blocker	5 (21.7%)	3 (13.0%)	0.67
Statin	1 (4.3%)	2 (8.7%)	1.00

ACEI/ARB, angiotensin-converting enzyme inhibitor/angiotensin receptor blocker; HVEI, high-voltage electrical injury

### Clinical, electrocardiographic and echocardiographic parameters

The comparisons of the clinical and the echocardiographic parameters are shown in Table 2. The baseline and six-week systolic and diastolic blood pressures and heart rates were higher in the HVEI group than in the control group, but no difference was found at the six-month follow-up.

**Table 2:** Influence of high-voltage electrical injury on clinical, electrocardiographic and echocardiographic parameters

		HVEI group		
		
	Control group (*n *= 23)	Baseline (*n *= 23)	Six weeks (*n *= 23)	Six months (*n *= 21)
Systolic blood pressure (mmHg)	119 ± 12	146 ± 14**	128 ± 11**	117 ± 10
Diastolic blood pressure (mmHg)	68 ± 12	84 ± 13**	77 ± 8**	68 ± 10
Heart rate (beats/min)	67 ± 7	80 ± 10**	77 ± 14**	70 ± 8
**Echocardiography**				
LVEF (%)	64 ± 4	66 ± 4	66 ± 5	64 ± 5
LVMI (g/m^2^)	89 ± 9	94 ± 13	92 ± 9	92 ± 8
E velocity (cm/sec)	71 ± 17	80 ± 17	69 ± 14	69 ± 19
A velocity (cm/sec)	66 ± 21	76 ± 18	71 ± 15	74 ± 19
Deceleration time (msec)	232 ± 75	199 ± 52	208 ± 38	234 ± 65
E/A ratio	1.2 ± 0.6	1.1 ± 0.3	1.0 ± 0.3	1.0 ± 0.5
Ea velocity (cm/sec)	9.3 ± 3.4	9.3 ± 2.7	8.2 ± 2.4	8.0 ± 2.1
E/Ea ratio	8.3 ± 3.0	9.1 ± 2.3	8.8 ± 2.3	8.9 ± 2.7

**P <0.01 vs. control group. A, late transmitral filling wave; E, early transmitral filling wave; Ea, medial mitral annulus wave; HVEI, high-voltage electrical injury; LVEF, left ventricular ejection fraction; LVMI, left ventricular mass index

The echocardiographic analysis showed that there were no differences between the control group and the HVEI group, including their LVEF, early transmitral filling wave velocity (E), late transmitral filling wave velocity (A), E/A ratio, deceleration time, early diastolic velocity of the medial mitral annulus (Ea) and E/Ea ratio during the study period. The average values of the S and SR of the longitudinal, circumferential and radial functions are presented in Table 3. The S data were comparable between the control group and the HVEI group throughout the study. However, the longitudinal, circumferential and radial SRs were significantly higher in the HVEI group at the baseline and at six weeks compared with the control group. However, at the six-month follow-up, there was no difference in the SRs between the HVEI group and the control group.

**Table 3:** Influence of high-voltage electrical injury on left ventricular function assessed with strain and strain rate

		HVEI group		
		
	Control group (*n *= 23)	Baseline (*n *= 23)	Six weeks (*n *= 23)	Six months (*n *= 21)
**Strain (%)**				
Longitudinal	-18.1 ± 1.6	-18.9 ± 3.7	-18.8 ± 3.4	-19.0 ± 2.8
Circumferential	-18.9 ± 3.4	-19.8 ± 5.5	-19.7 ± 3.6	-19.7 ± 5.2
Radial	53.3 ± 16.9	52.0 ± 17.1	46.7 ± 18.0	48.0 ± 14.6
**Strain rate (1/sec)**				
Longitudinal	-1.19 ± 0.18	-1.48 ± 0.39**	-1.36 ± 0.29*	-1.20 ± 0.21
Circumferential	-1.59 ± 0.37	-2.19 ± 0.91**	-1.90 ± 0.56*	-1.73 ± 0.56
Radial	1.97 ± 0.43	2.80 ± 0.19**	2.67 ± 0.98**	2.29 ± 0.87

*P <0.05 vs. control group; **P <0.01 vs. control group. HVEI, high-voltage electrical injury

### Vertical injury vs. horizontal injury

In this study, there were 17 patients who suffered from vertical current injury and 6 patients who exhibited horizontal current injury from the entrance to the exit sites. The peak levels of CK-MB and cTnI were not different between the patients with vertical injury and horizontal injury (76.14 ± 96.76 ng/ml vs. 121.97 ± 140.29 ng/ml, *P *= 0.39, respectively, and 0.10 ± 0.13 ng/ml vs. 0.04 ± 0.01 ng/ml, *P *= 0.07, respectively). In addition, the values of the echocardiography, S and SR showed no difference between the patients with vertical injury and horizontal injury at the baseline and at the six-month follow-up (Table 4).

**Table 4:** Influence of high-voltage electrical injury according to the pathway of the electrical current (vertical vs. horizontal)

	Baseline			Six months		
		
	Vertical axis (*n *= 17)	Horizontal axis (*n *= 6)	*P*-value	Vertical axis (*n *= 16)	Horizontal axis (*n *= 5)	*P*-value
LVEF (%)	66 ± 4	66 ± 3	0.92	64 ± 5	65 ± 5	0.60
E wave velocity (cm/sec)	84 ± 24	84 ± 13	0.96	70 ± 20	66 ± 16	0.65
A wave velocity (cm/sec)	77 ± 17	74 ± 23	0.75	75 ± 20	68 ± 15	0.49
Deceleration time (msec)	200 ± 48	198 ± 65	0.93	243 ± 70	207 ± 43	0.29
E/A ratio	1.1 ± 0.3	1.2 ± 0.4	0.44	1.0 ± 0.5	1.0 ± 0.4	0.96
Ea (cm/sec)	8.7 ± 2.6	10.9 ± 2.6	0.09	8.1 ± 2.3	7.8 ± 1.5	0.80
E/Ea ratio	10.1 ± 7.7	7.9 ± 1.4	0.08	9.1 ± 3.0	8.4 ± 1.3	0.63
**Strain (%)**						
Longitudinal	-19.0 ± 4.1	-18.7 ± 2.3	0.85	-19.2 ± 2.9	-18.5 ± 3.0	0.62
Circumferential	-19.3 ± 4.3	-21.2 ± 8.3	0.47	-19.6 ± 5.8	-19.8 ± 3.6	0.95
Radial	50.9 ± 17.8	55.0 ± 15.7	0.63	46.1 ± 15.5	54.0 ± 10.5	0.30
**Strain rate (1/sec)**						
Longitudinal	-1.50 ± 0.42	-1.44 ± 0.32	0.76	-1.22 ± 0.21	-1.15 ± 0.21	0.51
Circumferential	-2.08 ± 0.46	-2.52 ± 0.69	0.55	-1.82 ± 0.59	-1.45 ± 0.34	0.21
Radial	2.87 ± 1.19	2.59 ± 0.78	0.61	2.38 ± 0.95	1.98 ± 0.48	0.38

A, late transmitral filling wave; E, early transmitral filling wave; Ea, medial mitral annulus wave; LVEF, left ventricular ejection fraction.

## Discussion

The risk of developing chronic myocardial disability after HVEI is not well known. This study showed that, even after HVEI, the contractile performance of the myocardium was maintained if the LV systolic function was preserved immediately after HVEI.

The clinical presentation of myocardial damage by HVEI is variable and often missed because the victims rarely have typical chest pain, significant ECG changes or arrhythmia [5,10,11]. Previous studies demonstrated that the myocardial damage could occur after both low-voltage and high-voltage injuries [12,13]. However, evaluation of cardiac injury only on the basis of changes on ECG or CK-MB seems to be insufficient for determining the presence or the extent of myocardial injury after HVEI because of the non-specific characteristics and combined skeletal muscle damage [8]. Kerber *et al.* showed no demonstrable abnormalities of the LV contraction and perfusion functions after high-energy transthoracic shocks. The authors suggested that the diffuse nature of the intrathoracic current in transthoracic shock causes more patchy distribution of damage than focal necrosis. Therefore, functional sequelae of the myocardium may neither be detected with ultrasound nor radiolabeled microspheres [14]. The data may explain why a small number of patients manifested LV dysfunction after HVEI. We previously reported preserved LV systolic function by conventional echocardiography in survivors in the short term after HVEI [8]. In this study, we confirmed and extended our previous report, demonstrating that survivors of HVEI with preserved LVEF maintain their LV systolic function not only in the short term, but also in long-term follow-up.

In general, quantitative echocardiographic assessment of global LV function using the LVEF has several limitations [7,15]. It is well known that S is a measure of regional tissue deformation and that SR is a quantitative evaluation of deformation velocity, both of which are the primary parameters derived from tissue Doppler [7]. Recently, the S and the SR, which are based on the 2D speckle tracking method, have been shown to provide accurate and angle-independent measurements [16]. A quantitative technique identifying early functional abnormalities in patients with apparently normal LVEF has been proposed to detect subclinical LV dysfunction [17]. In this study, there were no differences in the S between the HVEI group and the control group during the study period. In terms of the SRs, the baseline and six-week follow-up SRs were significantly increased in the HVEI group compared with the control group, but there was no difference at the six-month follow-up. This could be explained by the SR correlating with the rate of change in the pressure that is used to reflect contractility and S being an analog of the regional ejection fraction [18]. Early stage HVEI is accompanied by excessive emotional and physical stress, and a large amount of catecholamine may be released that induces high blood pressure and increases the heart rate [19]. In this study, not only the blood pressure and heart rates were increased at the baseline and at the six-week follow-up in the HVEI group, the SRs of the HVEI group were also significantly increased compared with those of the control group. However, at the six-month follow-up, there was no difference in the blood pressure, heart rate and SR between the HVEI group and the control group. Voige *et al.* reported that increasing doses of dobutamine in normal myocardium are associated with increasing SR but that, in contrast, myocardial S shows a biphasic response, which initially increases and then decreases as the heart rate increases [20]. The patients in an early state of HVEI could be considered to be at the peak stage of dobutamine stress due to the massive release of catecholamine associated with severe psycho-physiological stress. Among the patients who were excluded from the study due to LV dysfunction, two of them exhibited characteristics of stress cardiomyopathy in their echocardiograms, which might be associated with high levels of circulating catecholamine [21]. In addition, many of the patients in this study underwent repeated reconstructive surgery around the time of the six-week follow-up. This could explain the increased SR and the minimal change in the S at the initial stage and at six weeks. In this study, we reconfirmed our previous reports that the SR increases in the early-stage of HVEI, compared with normal controls and that there were no differences in the S between the groups. In addition, we found that there was no difference in the SR and the S between the HVEI group and the control group at the six-month follow-up. Thus, even after HVEI, the contractile performance of the myocardium is well maintained if the LV systolic function is preserved immediately after HVEI.

There were some suggestions about myocardial damage according to the pathway of the electrical current in the patient's body, whether vertical or horizontal injury. Chandra *et al.* reported that the vertical pathway of the electrical current through the body is one of the early predictors of myocardial damage in patients with HVEI because the vertical pathway is likely associated with a longer tissue transit of electricity and greater tissue necrosis [5]. However, they identified myocardial damage only in patients in whom the CK-MB level was more than 3% greater than that of the total CK. Such a finding is quite non-specific when the victim has suffered severe skeletal muscle damage. It is to be expected that those who are exposed to vertical electrical injury and the associated increase in skeletal muscle and systemic damage have a higher mortality rate compared with patients with injuries localized to the upper extremities and trunk [14]. In this study, there was no difference in the CK-MB and cTnI levels between the patients with vertical and horizontal pathway injuries. In addition, we found no difference in the LVEF, S and SR between the vertical and horizontal pathway groups at baseline and at the six-month follow-up. Thus, the myocardium and the LV function might not be directly affected by the direction of the electrical current pathway in HVEI.

This study has several potential limitations. First, 41 out of 64 patients (64%) were excluded during the screening. Among these, 17 patients were excluded because they had visited our hospital more than 48 hours after HVEI, meaning we could not evaluate acute stages of the injury, including their cardiac enzymes, ECG and echocardiographic results. In addition, 12 patients did not participate in our study due to associated LV dysfunction, multi-organ failure or severe sepsis. Therefore, there could be a selection bias, whereby only patients with less severe myocardial damage were enrolled in the study. Second, in patients with HVEI, confusion, impaired recall and the loss of consciousness tend to be common [4]. Although, the duration of the high voltage electrical current exposure could be an important factor in determining the presence or absence of SR abnormalities, it is quite challenging to accurately estimate the duration of their exposure to the electrical current by history taking, and we were unable to address this issue. Third, after HVEI, there is a surge in stress hormones, such as catecholamine, and we assumed that the differences between the HVEI group and the control group in terms of their blood pressures, heart rates and SRs were associated with elevated stress hormones during the early stage after the HVEI. However, we did not check circulating biomarkers which represent the degree of the victim's stress. Finally, we conducted the last follow-up at six months after the HVEI. It remains unclear whether different results would have been obtained with an examination at a later follow-up date.

## Conclusions

In conclusion, the long-term contractile performance of the myocardium is preserved when patients do not experience LV dysfunction in the early stages after HVEI.

## Key messages

• The long-term contractile performance of the myocardium is preserved when patient do not experience left ventricular dysfunction in the early stages after high-voltage electrical injury.

• The myocardium and the left ventricular function might not be directly affected by the direction of the electrical current pathway in high-voltage electrical injury.

## Abbreviations

ASE: American Society of Echocardiography; CK-MB: creatine kinase MB; cTnI: cardiac troponin I; EA: early diastolic; HVEI: high-voltage electrical injury; LVEF: left ventricular ejection fraction; S: strain; SR: strain rate

## Competing interests

The authors declare that they have no completing interests.

## Authors' contributions

KHP and WJP were responsible for the conception and design of the study. SWC and SAK acquired the data. KHP, SJH and HSK analyzed and interpreted the data. KHP, SHJ, SHK and WJP drafted the manuscript. KHP, SJH and KHP critically revised the manuscript for important intellectual content. All authors read and approved the final manuscript for publication.
